# Analysis of Viscoelastic Behavior of Polypropylene/Carbon Nanotube Nanocomposites by Instrumented Indentation

**DOI:** 10.3390/polym12112535

**Published:** 2020-10-29

**Authors:** Felicia Stan, Adriana-Madalina Turcanu (Constantinescu), Catalin Fetecau

**Affiliations:** Center of Excellence Polymer Processing, Dunarea de Jos University of Galati, 47 Domneasca, 800 008 Galati, Romania; madalina.constantinescu@ugal.ro (A.-M.T.); fetecau.catalin@ugal.ro (C.F.)

**Keywords:** polypropylene, carbon nanotubes, indentation, shear creep function

## Abstract

In this work, the viscoelastic behavior of polypropylene (PP)/multi-walled carbon nanotube (MWCNT) nanocomposites was investigated by indentation testing and phenomenological modeling. Firstly, indentation tests including two-cycle indentation were carried out on PP/MWCNT nanocomposite with three MWCNT loadings (1, 3 and 5 wt %). Next, the Maxwell–Voigt–Kelvin model coupled with two-cycle indentation tests was used to predict the shear creep compliance function and the equivalent indentation modulus. The indentation hardness and elastic modulus of the PP/MWCNT nanocomposites extracted based on the Oliver and Pharr method were compared with the equivalent indentation modulus predicted based on the Maxwell–Voigt–Kelvin mode. The experimental results indicated that the addition of nanotubes into the polypropylene has a positive effect on the micro-mechanical properties of PP/MWCNT nanocomposites. Indentation hardness and elastic modulus increased significantly with increasing MWCNT loading. The creep resistance at the micro-scale of the PP/MWCNT nanocomposites improved with the addition of MWCNTs, with creep displacement reduced by up to 20% by increasing the carbon nanotube loading from 1 to 5 wt %. The Maxwell–Voigt–Kelvin model with three and five Voigt–Kelvin units accurately predicted the shear creep function and its change with increasing MWCNT loading. However, the equivalent indentation modulus was found to be sensitive to the number of Voigt–Kelvin units: the more Voigt–Kelvin units, the better the model predicts the equivalent indentation modulus.

## 1. Introduction

Polymer/carbon nanotube (CNT) nanocomposites are currently used in many engineering applications [1,2,3,4] and a lot of research has been devoted to investigating and improving the processing–structure–property relationships, which are summarized in several reviews [5,6,7,8,9,10,11,12,13]. Polymer/CNT nanocomposites are typically multiscale materials that are homogeneous in the macro-scale and heterogeneous in the micro- and nano-scales. Therefore, knowledge of the mechanical behavior of these nanocomposites at micro- and nano-scales is crucial to make them as efficient as possible.

Depth sensing indentation (DSI) or instrumented indentation (II) has been used to characterize the micro- and nano-mechanical behavior of various polymer/CNT nanocomposites [14,15,16,17,18,19,20,21,22,23,24,25,26]. DSI can improve the scientific understanding of polymer/CNT nanocomposites and provides essential information on near surface properties of components, such as the indentation hardness and elastic modulus [14,17,21], and creep [15,16,19,22,25]. In addition, DSI can be used to investigate filler-network heterogeneity [24,27,28,29] and distribution of crystalline and amorphous regions within semi-crystalline polymers [30,31].

While for conventional elastic-plastic materials there is a broad consensus about the design of indentation experiments and data analysis, considerable challenges remain for polymers and polymer-based nanocomposites, although the key issues are generally well documented [20,23,32,33,34,35,36,37,38,39,40,41,42]. This is due to the complexity of polymer matrix deformation during the indentation process. When a sharp indenter is used on viscoelastic materials to extract mechanical properties, both time-independent and time-dependent deformations occur, like time-independent elasto-plastic deformation during the loading stage, time-dependent viscoelastic or visco-plastic deformations during the initial part of the unloading curve, and time-dependent viscoelastic deformation during the holding stage. These deformations can lead to errors in the calculation of the mechanical properties (hardness and modulus) from the initial part of unloading curve since the assumption of fully elastic unloading is invalidated [33,34,36,37,40]. On the other hand, the plastic deformations during the initial part of the loading curve may affect the creep deformation during the holding stage.

Thus, over the years, several experimental and analytical solutions have been proposed to extract the mechanical properties of polymers, including the time-dependent properties, by removing or isolating the plastic deformations from the measured load-displacement curves. When plastic deformations are not significant and may be ignored, to account for the time-dependent deformation, i.e., to allow the material to “creep out”, a dwell period at the maximum load was introduced before the onset of unloading to obtain pseudo-elastic behavior on unloading [34]. However, inherent in this rationale is the fact that the creep rate will fall off with time, possibly because of a decreasing contribution from primary creep and the dwell time is not known a priori. Further, to deal with the time-independent and time-dependent processes during the indentation of polymers and to evaluate the viscoelastic properties from the experimentally measured load-displacement curve, several indentation techniques have been proposed. For example, Mencik et al. [43,44] proposed a procedure to determine the shear creep compliance of linear viscoelastic-plastic polymers based on a five-step indentation procedure. Although the indentation depth-time curves were predicted very well using the shear creep compliance function, it also contains the contribution of plastic deformation and, therefore, is not the true shear creep compliance. To separate the plastic deformation from the elastic and viscoelastic deformations Zhang et al. [45,46] also proposed a five-step indentation scheme that was coupled with an analytical solution for the elastic–viscoelastic deformation based on the concept of “effective indenters”. Similarly, a two-cycle indentation test was used to investigate the creep behavior of polymers [47,48] and polymer-based composites [49]. According to this approach, the effect of plastic deformation that occurs during the first loading-unloading indentation cycle is limited to the deformation of the surface of the indented half-space [47,48]. Therefore, the second reloading-holding-unloading cycle can be considered as an indentation creep test into a viscous material and the displacement-time curve can be used to determine the indentation creep displacement [47,48,49]. A study by Kato et al. [50] proposed a three-cycle indentation test to investigate whether the plastic deformation occurs or not after the first cycle. Peng et al. [51,52,53] introduced the so-called revised step-hold method, which assumes that during the fast loading the elastic-plastic deformation is dominant and the viscoelastic deformation can be neglected, and only the viscoelastic deformation occurs during the holding stage. The revised load-depth curves obtained through a three-step procedure, i.e., a step-hold-unload indentation method, were used in the phenomenological model to calculate the shear creep compliance [51,52,53].

To conclude, several experimental and analytical methodologies are available for investigating the time-dependent behavior of polymers and have already produced significant results, however their applicability to polymer/CNT nanocomposites remains to be investigated.

In this work, the viscoelastic properties of polypropylene (PP) filled with multi-walled carbon nanotubes (MWCNTs) are investigated by indentation (Vickers indenter) testing and the phenomenological model. Firstly, to fully assess the behavior of PP/MWCNT nanocomposites with three MWCNT loadings (1, 3, and 5 wt.%), three indentation tests, i.e., loading-unloading, loading-holding-unloading, and two cycle loading-unloading and reloading-holding-unloading were carried out. Secondly, the time-dependent indentation displacement from the two-cycle indentation tests was analyzed within an analytical framework using the Maxwell–Voigt–Kelvin phenomenological model. Next, the mechanical properties (i.e., indentation hardness and elastic modulus) were extracted using the standard Oliver and Pharr (O and P) method [32] and compared with the equivalent indentation modulus predicted by the Maxwell–Voigt–Kelvin model. Finally, the elastic modulus, indentation hardness, and creep resistance are discussed in terms of MWCNT loading.

## 2. Materials and Methods

### 2.1. Materials and Manufacturing

The nanocomposites under investigation are based on polypropylene (PP, ExxonMobil Chemical, Huston, TX, USA) and 1, 3, and 5 wt % of multi-walled carbon nanotubes (NC7000^TM^, Nanocyl S.A., Sambreville, Belgium [54]) The PP/MWCNT nanocomposites with 1, 3 and 5 wt % of MWCNTs were purchased in the form of pellets from Nanocyl (Sambreville, Belgium). The PP/MWCNT pellets were injection-molded into standard dog-bone specimens (according to ISO 527 type 1B) using the following injection molding parameters: melt temperature of 215 °C, mold temperature of 40 °C, injection pressure of 80 MPa, holding pressure of 65 MPa, holding time of 35 s, and cooling time of 25 s. Additional details on the injection molding of the PP/MWCNT nanocomposites can be found in [55].

### 2.2. SEM Analysis

To investigate the dispersion of nanotubes within the PP matrix, scanning electron microscopy images were obtained using scanning electron microscope microscopy (Quanta 200, FEI, Hillsboro, OR, USA) at 25 kV accelerating voltage. Samples for SEM analysis were cryo-fractured under liquid nitrogen and attached to an aluminum sample holder using a double-stick electrically conductive carbon tape.

### 2.3. Indentation Experiments

**Figure 1:**
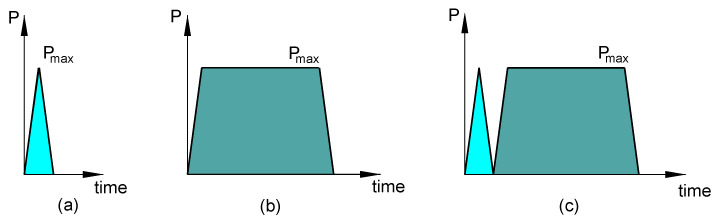
Schematic representation of the indentation loading profile for (**a**) 2-step, (**b**) 3-step, and (**c**) 2-cycle indentation tests.

Indentation tests were performed with a Micro-Combi Tester as part of an Indentation Platform (Micro-Combi Tester and NHT, Anton Paar GmbH, Graz, Austria). A Vickers diamond indenter tip with the angle between the axis of the diamond pyramid and its faces of 68° was employed. The diamond indenter has an elastic modulus of 1141 GPa and Poisson ratio of 0.07. To fully characterize the behavior of the PP/MWCNT nanocomposites, three indentation protocols were considered, as shown in Figure 1:

(a) 2-step indentation test (Figure 1a) that consists of linear loading to 1000 mN and unloading back to zero with the same rate of 2000 mN/min.
(b) 3-step indentation test (Figure 1b) that consists of loading, holding, and unloading. Both loading and unloading steps were carried out with the same rate of 2000 mN/min. A holding step of 40 s at constant force of 1000 mN was added at the end of the loading step. The 3-step indentation test is carried out in order to evaluate the mechanical properties based on the O and P method [32].
(c) 2-cycle indentation test (Figure 1c), which combines a 2-step indentation test with loading and unloading at the same rate (2000 m N/min) with a 3-step indentation test (loading/unloading with 2000 N/min, and a 300 s holding at 1000 mN). The interval time between the two cycles was set to 0 s and the tests were carried out on the same surface position.

All indentation experiments were performed at room temperature (23 °C) without a priori knowledge of the MWCNT distribution and dispersion. For each nanotube wt % and indentation protocol, ten to fifteen indents were performed on random locations to assess the repeatability of the measurements and to calculate the average value of the mechanical properties.

## 3. Results and Discussion

### 3.1. Morphology of PP/MWCNT Nanocomposites

**Figure 2:**
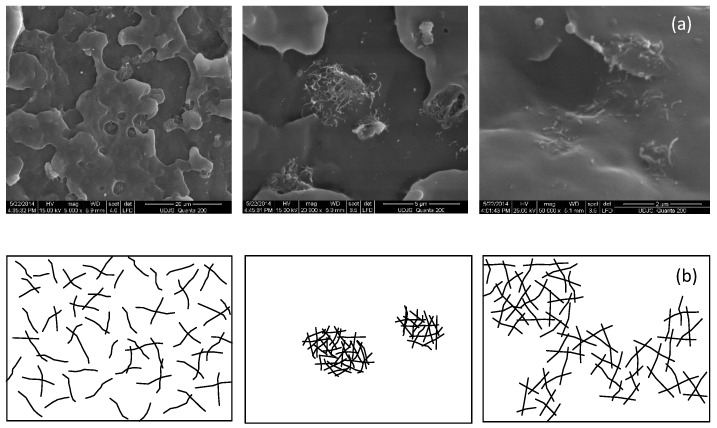
SEM images of the polypropylene (PP)/multi-walled carbon nanotubes (MWCNT) nanocomposite with 5 wt % (**a**) and schematic representation of MWCNTs (**b**).

Figure 2a shows the SEM images for the PP/MWCNT nanocomposite with 5 wt % of MWCNTs at three different magnification levels. It can be seen that the nanocomposite is apparently homogeneous in the macro-scale and heterogeneous in the micro-scale. Due to the van der Waals interactions between carbon nanotubes, clustering and tangling between MWCNTs were observed, as illustrated in Figure 2a. It should be mentioned that the same morphology was observed in the PP/MWCNT nanocomposites with 1 and 3 wt % (also analyzed but not displayed here), except that the size and the density of the agglomerates increased with increasing MWCNT loading. Based on the SEM imagines, three distributions were identified, as schematically represented in Figure 2b: (i) uniformly dispersed MWCNTs (with external diameter of about 20–40 nm), (ii) MWCNT spherical aggregates/agglomerations (up to 5 µm), leading to an abrupt transition to the neat matrix, and (iii) MWCNT loosely agglomerates.

### 3.2. Load Displacement Curves

**Figure 3:**
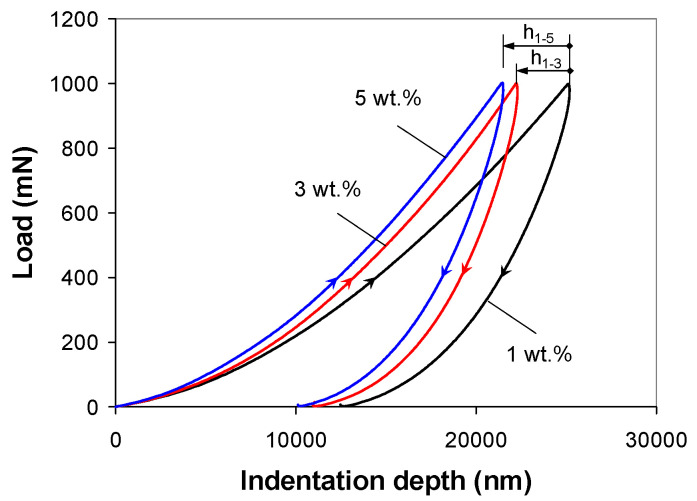
Experimental load–displacement response for 2-step indentation on the PP/MWCNT nanocomposites at 2000 mN/min loading/unloading rate.

Figure 3 shows the average indentation response, e.g., the P-h curves, of the PP/MWCNT nanocomposites at 2000 mN/min loading/unloading rate, for the 2-step indentation test. As can be seen in Figure 3, the initial part of the unloading curves displays the so-called “nose”. This response indicates the deformation of the PP/MWCNT nanocomposite due to the presence of creep at a rate greater than the elastic recovery [33,34], which invalidates the assumption of fully elastic unloading. However, the “nose” effect is obviously smaller in the nanocomposite with 5 wt % as compared with that with 1 wt %, indicating that this effect is rooted in the deformation of the polymer matrix and, on the other hand, the constraining effect of MWCNTs on the movement of polymer chains [11]. It can be seen that when the load reached the maximum load, the corresponding indentation depth decreases about 17%, from 25.185 µm to 21.499 µm with increasing nanotube loading from 1 to 5 wt %, indicating the reinforcement effect of the MWCNTs (Figure 3, h_1-3_ = 2.95 µm and h_1-5_ = 3.686 µm).

**Figure 4:**
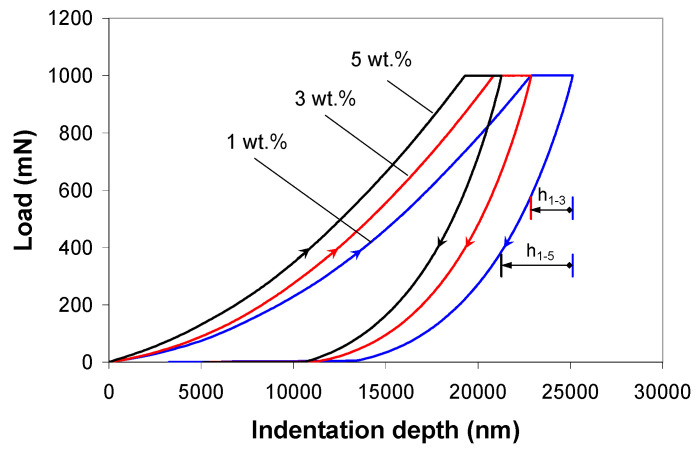
Experimental load-displacement response for 3-step indentation on the PP/MWCNT nanocomposites at 2000 mN/min loading/unloading rate and 40 s holding time.

The average indentation response of the PP/MWCNT nanocomposites during the 3-step indentation test is illustrated in Figure 4. As shown in this figure, the unloading curve does not display the “nose” effect since the nanocomposites were allowed to creep out 40 s at the maximum load. By increasing the nanotube loading, the curves shifted to lower indentation depths, indicating that the addition of nanotubes increases the stiffness and hardness of the PP/MWCNT nanocomposites. The maximum indentation depth decreased by approximately 10% (h_1-3_ =2.252 µm) when the nanotube loading increased from 1 to 3 wt % and 20% (h_1-5_ =2.862 µm) when the nanotube loading increased from 1 to 5 wt %, as shown in Figure 4.

**Figure 5:**
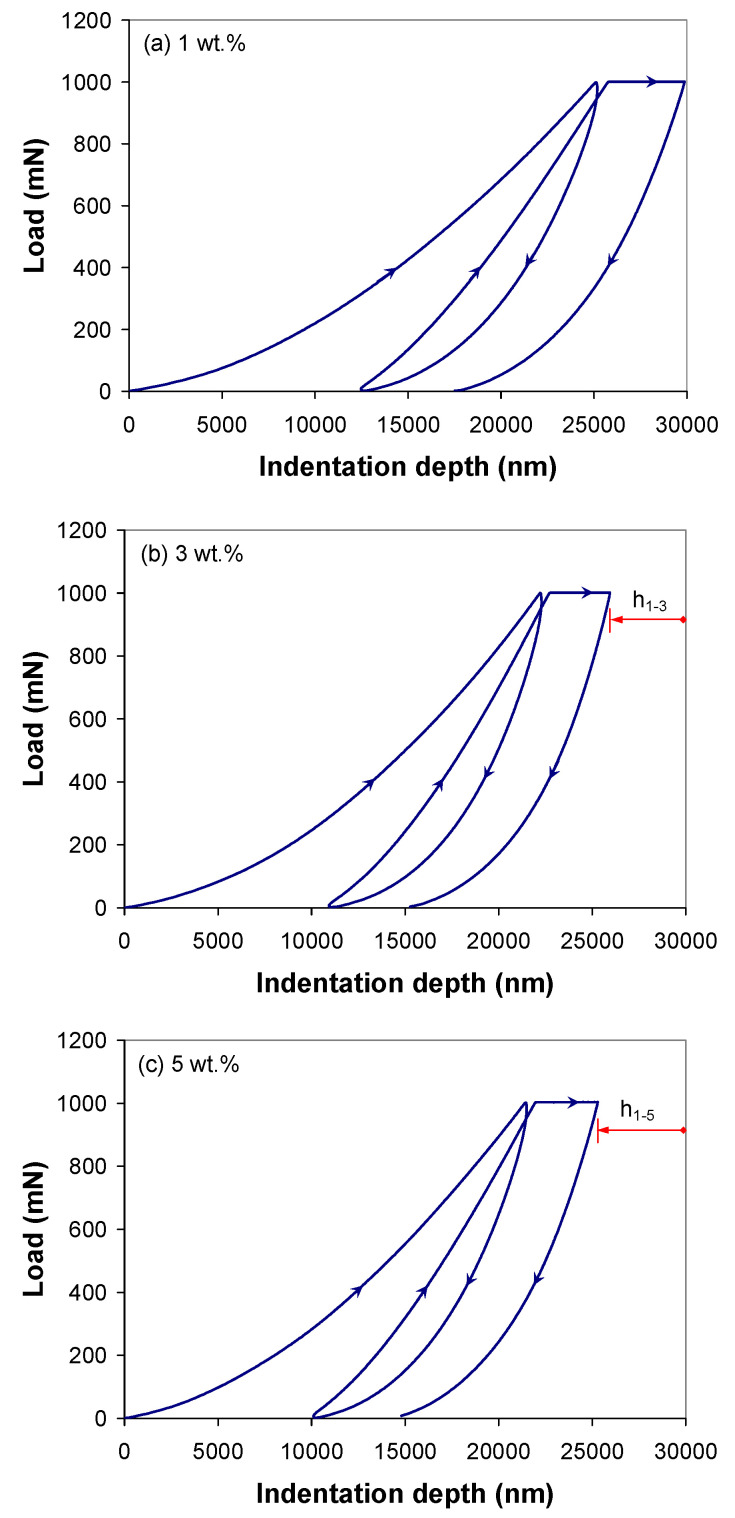
Experimental load-depth curves for 2-cycle indentation on the PP/MWCNT nanocomposites at 2000 mN/min loading/unloading rate and 300 s holding time.

Figure 5 shows the average P-h curves for the PP/MWCNT nanocomposites from the 2-cycle indentation test. As expected, the unloading curve of the first indentation cycle features the “nose” effect during the early stage of unloading. However, the unloading curve of the second indentation cycle indicates that the viscoelastic deformation is dominant during the second indentation cycle. As the nanotube loading increased from 1 to 5 wt %, the maximum indentation depth decreased by about 20%, as shown in Figure 5 (h_1-3_ = 3.933 µm and h_1-5_ =4.579 µm).

### 3.3. Indentation Modulus and Hardness

The traditional O and P method [32] was applied for analyzing the experimental indentation load-displacement curves from the 3-step indentation tests using the Indentation software (Anton Paar GmbH, Graz, Austria). The indentation modulus was obtained from the unloading part of the 3-step indentation load-displacement curves shown in Figure 4, under the assumption that the early stage of the unloading is elastic [32]. The indentation hardness and modulus were calculated using Equations (1) and (2), respectively,

(1) $$H=\frac{P_{max}}{A_{p}},$$

(2) $$\frac{1}{E_{R}}=\frac{1-\nu^{2}}{E_{IT}}+\frac{1-\nu_{i}^{2}}{E_{i}}$$

where *P*_max_ is the maximum applied load, *A_p_* is the projected contact area, *E_R_* is the reduced contact modulus between the tip and the sample, ν and ν*_i_* are the Poisson ratio of the sample and the diamond tip, respectively, and *E_i_* is the Young modulus of the diamond tip.

Table 1 summarizes the indentation hardness, *H_IT_*, and elastic modulus, *E_IT_*, of the PP/MWCNT nanocomposites as a function of MWCNT loading. Each value in Table 1 represents the average of at least ten indentations along the melt flow direction of the injection-molded samples. The mechanical properties extracted at different locations along the melt flow direction have good repeatability, with the standard deviation less than 1% and 9% for *E_IT_* and *H_IT_*, respectively.

As can be seen in Table 1, the presence of nanotubes within the PP matrix led to changes in micro-mechanical properties of the nanocomposites: both indentation modulus and hardness steadily increased as nanotube loading increased. However, the enhancement in the indentation hardness is more significant. Upon increasing MWCNT loading from 1 to 5 wt %, the indentation hardness of the PP/MWCNT nanocomposite increased by about 43%, while the elastic modulus by only 28.5%. The increase in mechanical properties can be correlated with the formation and consolidation of the carbon nanotube networks with increasing MWCNT loading. On the other hand, the increase in the indentation hardness can be explained by the formation of a highly oriented skin layer leading to increased stiffness and therefore a reinforcing effect [55,56,57]. It has been reported that for the injection-molded samples, the skin layer has a highly oriented structure with a “strengthening” feature, while the core layer has a random crystalline structure with a “toughening” feature [55,56,57].

### 3.4. Creep of PP/MWCNT Nanocomposites

#### 3.4.1. Indentation Creep Displacement

In this work, to extract the creep indentation displacement, the 2-cycle indentation tests were considered instead of the standard 3-step indentation tests. One feature of the 2-cycle indentation test is that the indentation-induced plastic deformation during loading can be minimized if not canceled [46,47,49]. It was argued that the plastic deformation, which occurs during the first indentation cycle of the test—loading and unloading, leads to self-equilibrated residual stresses within the indentation-half space [46,47]. Therefore, in the absence of the residual stresses, the effect of plastic deformation on the second indentation cycle is limited to the deformation of the surface of the indented half space [46,47]. Thus, the second cycle can be considered as an indentation creep test in a viscous material, which undergoes only viscoelastic deformations [46,47,49].

**Figure 6:**
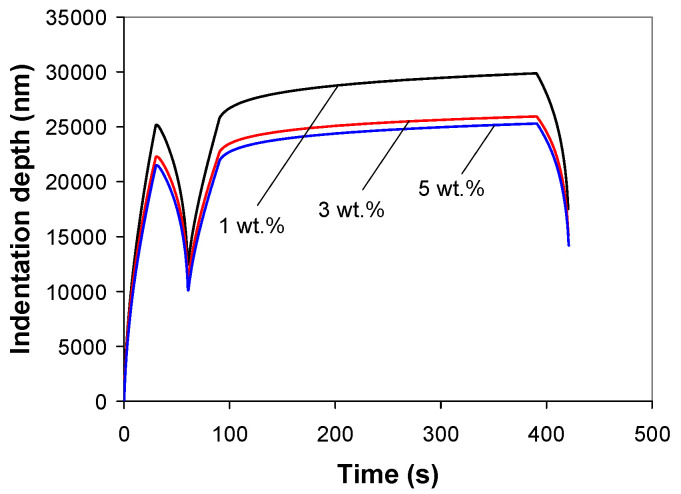
Displacement-time curves from 2-cycle indentation on the PP/MWCNT nanocomposites at 2000 N/min loading/unloading rate and 300 s holding time.

Figure 6 shows the experimental indentation displacement versus time curves from the 2-cycle indentation tests. It is evident that the indentation depth during the holding stage varies with MWCNT loading, i.e., the creep displacement decreases with increasing nanotube wt %. The sharp drop in creep displacement with increasing nanotube loading from 1 to 3 wt % is followed by a moderate reduction in the creep displacement for further increase of MWCNT loading (5 wt %).

**Figure 7:**
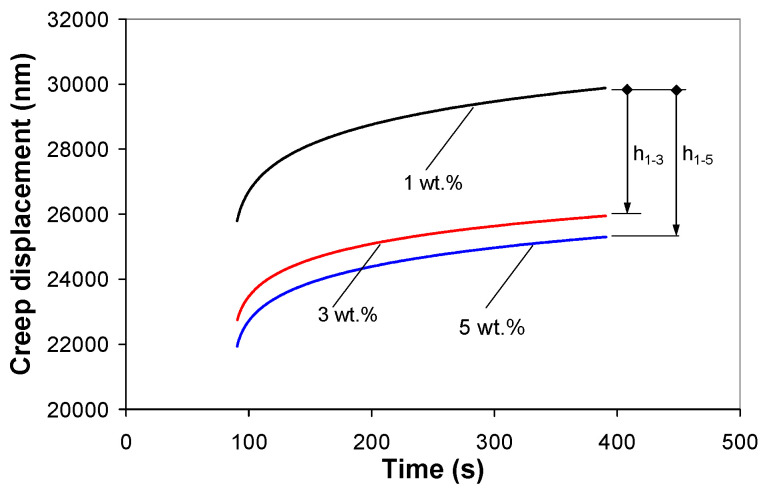
Indentation creep displacement curves for the PP/MWCNT nanocomposites (holding stage of the 2-cycle indentation test).

The creep displacement curves (average curve over at least five indents) from the holding stage of the 2-cycle indentation test are shown in Figure 7. The analysis of the creep curves reveals that the addition of nanotubes significantly improved the creep behavior of the PP. The final creep displacements were 29.882, 25.943, and 25.288 µm respectively for 1, 3, and 5 wt %, respectively. Relative to the nanocomposite with 1 wt %, incorporation of 3 wt % of MWCNTs into the PP polymer decreased the creep displacement after 300 s by 13% (the corresponding decrease in displacement is *h*_1-3_ = 3.940 µm) while the creep displacement decreases by 15% with the addition of 5 wt % of MWCNTs (the corresponding decrease in displacement is *h*_1-5_ = 4.582 µm).

Generally, the creep response of the polymer/CNT nanocomposites is a combined effect of the morphological changes, such as orientation and crystallization due to the presence of carbon nanotubes, and their nano-effects, such as aspect ratio and interfacial bonding between nanotubes and polymer matrix. As is well known, the presence of filler leads to chain immobilization [58,59,60], while a good filler-matrix interfacial bonding results in inhibiting chain disentanglement, stretching and fragmentation of the macromolecules [61,62]. At low nanotubes loading, the polymer matrix is not reinforced sufficiently and highly localized strains can occur in the matrix, breaking the bond between the matrix and nanotubes, leaving the matrix weakened by non-reinforcing de-bonded nanotubes. At higher nanotubes loading, the confinement effect of the nanotube networks prevents the deformation of the polymer chains. The nanotubes act as confinement barriers, limiting the movement of the PP chains when subjected to an external indentation force, hence, they restrict the viscous flow of the amorphous region. The crystallinity of the injection-molded PP/MWCNT nanocomposite only slightly increased with increasing MWCNT loading (38.32, 39.22, and 40.06% for 1, 3, and 5 wt %, respectively) as shown in [55].

#### 3.4.2. Creep Compliance Function

To describe the viscoelastic behavior of polymers and their nanocomposites and to obtain the corresponding time-dependent displacement or creep compliance function, the concept of hereditary integral operator and the elastic-viscoelastic correspondence principle [63,64,65,66] have been applied, and different approaches were developed to model the indentation into a linearly viscoelastic half space [20,35,39,49,50,51,52,53,56,67].

The general relationship between the time-dependent indentation displacement and load can be written as [39,50,51,63,64,65,67,68]

(3) $$h^{\left(n+1\right)/n}\left(t\right)=\frac{1-\nu }{4C_{n}}\int_{0}^{t}J\left(t-s\right)\frac{dP}{ds}ds,$$

where J(t) is the creep compliance function in shear, ν is the Poisson’s ratio, and n and $C_{n}$ are constants related to the geometry of the indenter tip.

Under step loading, for a sharp tip indenter (*n* = 1, $C_{1}=\tan\alpha /\pi$), based on the Equation (3), the indentation displacement can be written as

(4) $$h^{2}\left(t\right)=\frac{\pi \left(1-\nu \right)P_{max}}{4\tan\alpha }(t),$$

where *P*_max_ is the maximum indentation holding load and α is the included half-angle of the indenter.

**Figure 8:**
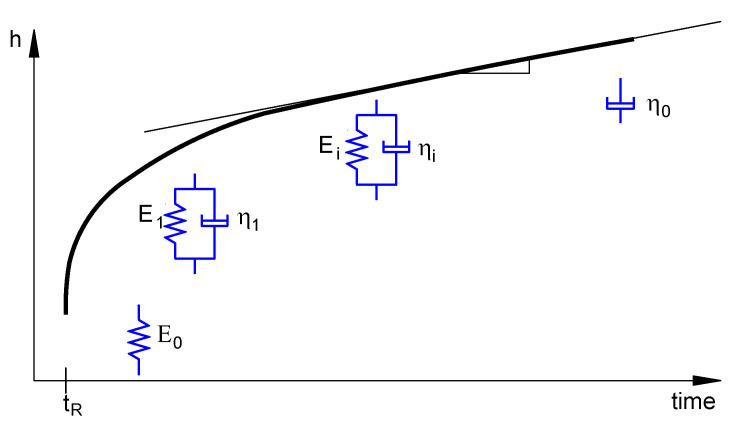
Schematic representation of the creep displacement with the Maxwell–Voigt–Kelvin model.

The choice of the *J*(*t*) function in Equation (4) provides a wide range of possibilities for modeling the indentation creep displacement. In the present work, the generalized Maxwell–Voigt–Kelvin model, as illustrated in Figure 8, was considered for which the shear creep compliance function is described by the following equation [43,49,53]:

(5) $$J\left(t\right)=J_{0}+\overset{N}{\underset{i=1}{\sum }}J_{i}\left[1-\exp\left(1-\frac{t}{\tau_{i}}\right)\right]+J_{v}t,$$

where $J_{0}$, $J_{v}$ and $J_{i}$ are the compliance constants, and $\tau_{i}$ are the retardation time constants. Note that the time–dependent irreversible viscous deformation is considered by the dashpot of viscosity η_0_.

Since step loading is very difficult to achieve, it is more realistic to consider a ramp loading. When a ramp loading is considered, substituting Equation (5) into Equation (3), after some manipulation, the indentation creep displacement at the peak of the ramp load can be expressed as [49]

(6) $$h^{2}\left(t\right)=\frac{\gamma^{2}}{\pi \tan\alpha }kt_{R}\left(J_{0}+J_{v}\left(t-\frac{t_{R}}{2}\right)+\sum_{i=1}^{n}J_{i}\left[1-\exp\left(\frac{-t}{\tau_{i}}\right)\rho_{i}\right]\right),\;\mathrm{for}\;t\geq t_{R},$$

in which *t*_R_ is the time corresponding to the ramp loading, k is the loading rate, γ = π/2, and $\rho_{i}$ is the ramp correction factor,

(7) $$\rho_{i}=\frac{\tau_{i}}{t_{R}}\left[\exp\left(\frac{t_{R}}{\tau_{i}}\right)-1\right],$$

which accounts for the difference in creep caused by the ramp loading when comparing with a step loading [49,69].

To accurately evaluate the creep compliance function, only the viscoelastic indentation displacement must be used in Equation (6) because the plastic deformation that occurs during the loading segment may considerably affect the indentation displacement. Thus, in this work, the viscoelastic displacement was obtained from the 2-cycle indentation procedure, as shown in Figure 7, and fitted into Equation (6) with three and five Voigt–Kelvin (VK) units. It is to be noted that the viscous element of the Maxwell model was disregarded, thus the model is coded as S + *n*VK.

The viscoelastic parameters were found from the best-fit using a nonlinear least-squares optimization algorithm (iterative Levenberg–Marquardt method [49,70]). The elastic parameter (*J*_0_) and the viscoelastic ($J_{i}$, $\tau_{i}$) parameters are listed in Table 2. Substituting viscoelastic parameters in Table 2 into Equation (5), one can obtain the shear creep compliance function for the PP/MWCNT nanocomposites and further predict the creep indentation displacement as in Equation (6). The creep displacement obtained from the S + *n*VK models was compared with the experimental data based on the root mean square error (RMSE) and the mean percentage error (MPE).

**Figure 9:**
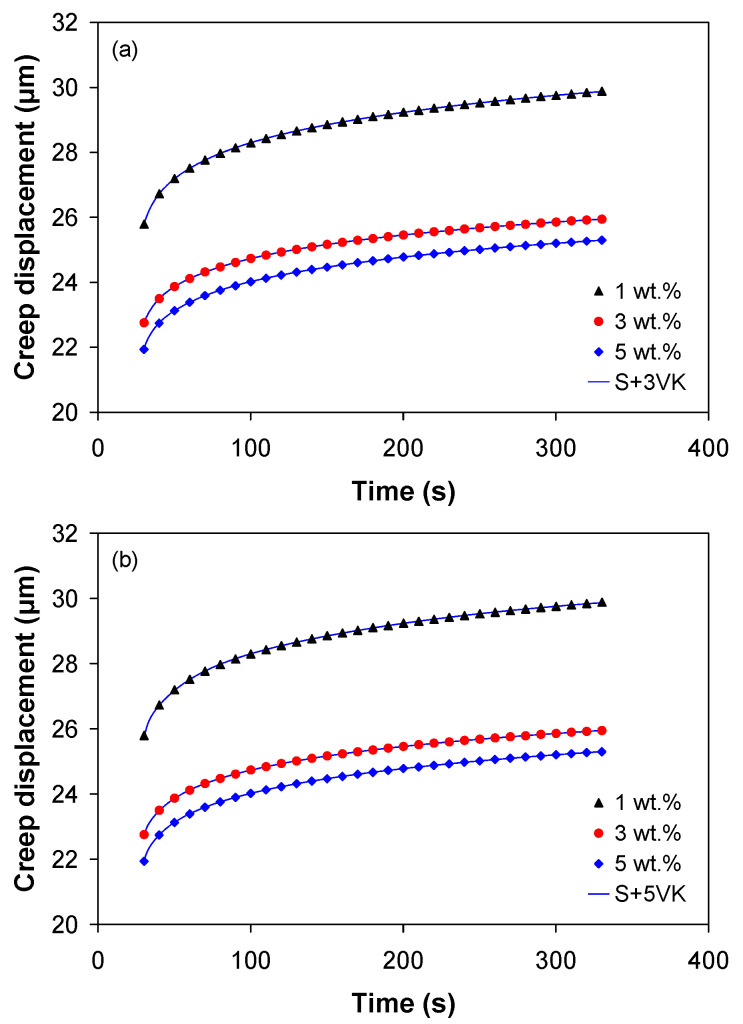
Comparison between experimental creep displacement and prediction based on the S + nVK model with (**a**) three VK units and (**b**) five VK units (predictions are shown as solid lines).

Figure 9 shows the experimental creep displacement data (filled symbols) and the values predicted by the S + *n*VK model (solid lines) with three and five VK units. Despite its simplicity, the model is capable of representing the main features of the creep displacement during the holding segment of the 2-cycle indention test. For all nanotube weight fractions, the predicted curves are in very good agreement with the experimental creep displacement data, as shown in Figure 9. The S + *n*VK model (*n* = 3 and 5) was found to produce a highly accurate fit, as shown in Table 2, with the *MPE* and *RMSE* less than 0.01% and 0.01, respectively.

The instantaneous elastic compliance *J*_0_ represents the value of instantaneous shear creep compliance at initial time. As shown in Table 2, the *J*_0_ value decreased with increasing nanotube loading indicating that the PP/MWCNT nanocomposite became more rigid and has greater indentation hardness at higher nanotube loading.

The instantaneous shear modulus and the shear modulus at infinite, respectively, were calculated from the parameters in Table 2, according to:

(8) $$G_{0}=1/\left(2J_{0}\right),$$

(9) $$G_{\infty }=2/(J_{0}+\sum_{i=1}^{N}J_{i}).$$

Table 3 shows that the $G_{0}$ modulus increases with increasing the number of VK units and reaches a steady state value for five VK units, while the $G_{\infty }$ modulus is almost independent of the number of VK units.

Table 4 gives the indentation modulus, *E_IT_*, the plane strain modulus, *M* = *E_IT_*/(1 − ν^2^), and the equivalent indentation modulus, 4*G*_0_, calculated based on the instantaneous shear modulus from Equation (8). Recall that that the *E_IT_* of the sample was calculated based on the O and P method [32] using the reduced modulus extracted from the 3-step indentation tests, as shown in Section 3.3.

From Table 4, it is observed that the more VK units are used, the better the S + *n*VK model predicts the equivalent indentation modulus. The equivalent indentation modulus predicted based on the S + three VK model is smaller than the plane strain modulus; about 60% of the plane strain modulus. However, when using five VK units, the equivalent indentation modulus is only slightly smaller as compared with the plain strain modulus (about 13, 9, and 6% for nanotube loading of 1, 3, and 5 wt %, respectively). On the other hand, it is observed that the 4*G*_0_ modulus increases (e.g., the immediate creep deformation decreases) with increasing MWCNT wt %.

## 4. Conclusions

In this work, the viscoelastic properties of the PP/MWCNT nanocomposites with three MWCNT loadings (1, 3, and 5 wt %) were investigated using 2-cycle indentation testing and the Maxwell–Voigt–Kelvin model. The main conclusions of this research are as follows:

- At micro-scale level, the addition of nanotubes into the polypropylene has a positive effect on the mechanical properties of the PP/MWCNT nanocomposites. The indentation hardness and elastic modulus increased significantly with increasing MWCNT loading, without statistically significant differences among different regions.
- The creep resistance of the PP/MWCNT nanocomposites improved with the addition of MWCNTs, with creep displacement reduced by up to 20% with increasing MWCNT loading from 1 to 5 wt %. It is suggested that the creep deformation of the PP/MWCNT nanocomposites would proceed by deformation of the matrix and the role of the nanotubes would be to increase the creep resistance by restricting the polymer chain movement.
- The Maxwell–Voigt–Kelvin model accurately predicted the shear creep indentation behavior of PP/MWCNT nanocomposites and its change with increasing MWCNT loading, providing an indirect method to estimate the equivalent indentation modulus. However, the equivalent indentation modulus was found to be sensitive to the number of Voigt–Kelvin units: the more Voigt–Kelvin units used, the better the model predicts the equivalent indentation modulus.

Two-cycle indentation testing together with the phenomenological model is a convenient and effective tool to characterize the viscoelastic behavior of polymer/CNT nanocomposites. Future experimental work will be directed towards investigating this hybrid experimental-analytical tool in relation to other parameters such as loading/unloading rate, maximum load, polymer matrix, etc.

## Figures and Tables

**Table 1:** Indentation properties of the PP/MWCNT nanocomposites.

MWCNTs (wt %)	H_IT_ (MPa)	E_IT_ (GPa)
1	85.33 (± 3.58)	1.71 (± 0.05)
3	99.52 (± 8.22)	1.99 (± 0.09)
5	122.09 (± 5.28)	2.21 (± 0.05)

**Table 2:** Best fitting viscoelastic parameters for the Maxwell–Voigt–Kelvin model.

Number of VK Units		1 wt %		3 wt %		5 wt %	
	*i*	*J_i_* (MPa)	τ*_i_* (s)	*J_i_* (MPa)	τ*_i_* (s)	*J_i_* (MPa)	τ*_i_* (s)
3	0	1.757	-	1.439	-	1.234	-
	1	0.505	5.70	0.360	6.77	0.405	4.92
	2	0.685	30.89	0.251	36.18	0.266	28.30
	3	0.374	235.54	0.450	252.97	0.474	223.29
	SSq	1.61 × 10^−1^		1.07 × 10^−1^		1.39 × 10^−1^	
	RMSE	0.004		0.003		0.003	
	MPE (%)	1.03 × 10^−2^		9.2 × 10^−3^		1.12 × 10^−2^	
5	0	1.198	-	1.003	-	0.878	-
	1	0.950	2.09	0.589	1.28	0.584	1.42
	2	0.444	22.69	0.273	11.96	0.275	10.89
	3	0.693	201.20	0.219	48.68	0.210	43.26
	4	1.04 × 10^−4^	200.05	6.53 × 10^−7^	199.63	0.111	194.59
	5	6.28 × 10^−5^	300.04	0.443	301.26	0.366	321.70
	SSq	6.58 × 10^−1^		1.31 × 10^−1^		1.07 × 10^−1^	
	RMSE	0.007		0.003		0.003	
	MPE (%)	2.02 × 10^−2^		9.96 × 10^−3^		9.24 × 10^−3^	

**Table 3:** Instantaneous shear modulus and shear modulus at infinite for the PP/MWCNT nanocomposite.

Number of VK Units	Modulus (GPa)	MWCNTs		
		1 wt %	3 wt %	5 wt %
3	*G*_0_	0.285	0.348	0.405
	*G*_∞_	0.602	0.800	0.841
5	*G*_0_	0.417	0.499	0.570
	*G*_∞_	0.609	0.792	0.825

**Table 4:** Indentation moduli for PP/MWCNT nanocomposites.

MWCNTs wt %	Methodology			
	S + *n*VK Model		Oliver and Pharr [32]	
	4*G*_0_ (GPa)		*E_IT_* (GPa)	*M* (GPa)
	S + 3VK	S + 5VK		
1	1.14	1.67	1.71 (±0.05)	1.91
3	1.39	1.99	1.99 (±0.09)	2.19
5	1.62	2.28	2.21 (±0.05)	2.43
